# Screening for depression in the general population through lipid biomarkers

**DOI:** 10.1016/j.ebiom.2024.105455

**Published:** 2024-11-20

**Authors:** Anna Tkachev, Elena Stekolshchikova, Anastasia Golubova, Anna Serkina, Anna Morozova, Yana Zorkina, Daria Riabinina, Elizaveta Golubeva, Aleksandra Ochneva, Valeria Savenkova, Daria Petrova, Denis Andreyuk, Anna Goncharova, Irina Alekseenko, Georgiy Kostyuk, Philipp Khaitovich

**Affiliations:** a Vladimir Zelman Center for Neurobiology and Brain Rehabilitation, Skolkovo Institute of Science and Technology, Moscow, 121205, Russia; b LLC NeurOmix, Moscow, 119571, Russia; c Mental-health Clinic No. 1, Named After N.A. Alekseev, Moscow, 117152, Russia; d Department of Basic and Applied Neurobiology, V. Serbsky Federal Medical Research Centre of Psychiatry and Narcology, 119034, Moscow, Russia; e Economy Faculty, M.V. Lomonosov Moscow State University, 119991, Moscow, Russia; f Moscow Center for Healthcare Innovations, Moscow, 123473, Russia; g Shemyakin-Ovchinnikov Institute of Bioorganic Chemistry, Russian Academy of Science, Moscow Region, 142290, Russia

**Keywords:** Lipids, Biomarkers, Depression, Lipidomics, Blood plasma

## Abstract

**Background:**

Anxiety and depression significantly contribute to the overall burden of mental disorders, with depression being one of the leading causes of disability. Despite this, no biochemical test has been implemented for the diagnosis of these mental disorders, while recent studies have highlighted lipids as potential biomarkers.

**Methods:**

Using a streamlined high-throughput lipidome analysis method, direct-infusion mass spectrometry, we evaluated blood plasma lipid levels in 604 individuals from a general urban population and analysed their association with self-reported anxiety and depression symptoms. We also assessed lipidome profiles in 32 patients with clinical depression, matched to 21 healthy controls.

**Findings:**

We found a significant correlation between lipid abundances and the severity of self-reported depression symptoms. Moreover, lipid alterations detected in high scoring volunteers mirrored the lipidome profiles identified in patients with clinical depression included in our study. Based on these findings, we developed a lipid-based predictive model distinguishing individuals reporting severe depressive symptoms from non-depressed subjects with high accuracy.

**Interpretation:**

This study demonstrates the possibility of generalizing lipid alterations from a clinical cohort to the general population and underscores the potential of lipid-based biomarkers in assessing depressive states.

**Funding:**

This study was sponsored by the Moscow Center for Innovative Technologies in Healthcare, №2707-2, №2102-11.

**Research in context**

**Evidence before this study**

Blood lipids have demonstrated an unusually strong association with psychiatric disorders among various molecular phenotypes examined, such as polar metabolites, gene expression and genetic information. Studies directly investigating the blood plasma lipidome composition in depression and other psychiatric disorders have revealed significant alterations in patients when compared to healthy controls.

**Added value of this study**

In this study, we evaluate the association between self-reported anxiety and depression symptoms from an urban population and the blood lipidome. We show that depressive symptoms, in particular, are correlated with certain blood lipid levels, and that lipidome alterations from high-functioning individuals from the general population with pronounced depression scores mirror those of clinically depressed patients, which is an important step towards a clinically applicable molecular test for depression risk assessment.

**Implications of all the available evidence**

Blood lipids show consistent alterations in psychiatric disorders, and depression in particular, in both clinical patients and general population samples, making them promising candidates for molecular testing of mental disorders.

## Introduction

Anxiety and depression significantly contribute to the global burden of mental disorders, with an estimated worldwide population prevalence of approximately 12% and 6% experiencing a respective episode within the past year.^1^, ^2^, ^3^ Depression in particular, is one of the leading causes of disability worldwide.^4^^,^^5^ Although lifestyle and social factors are believed to influence the onset of these diseases, there is also an association with specific genetic risk factors and epigenetic molecular changes.^4^^,^^6^ Given the substantial evidence of intrinsic biological processes associated with psychiatric disorder development, scientists are exploring potential molecular markers to supplement current interview-based diagnostic methods. Promising results have been found for depression and, to a lesser extent, anxiety disorders.^7^, ^8^, ^9^, ^10^, ^11^, ^12^, ^13^, ^14^

Several computational models based on blood biomarkers have been proposed for predicting depressive states. For instance, a study involving 897 subjects affected by the Great East Japan Earthquake suggested the potential for categorizing individuals with high levels of depressive symptoms based on their blood plasma metabolite profiles.^15^ Blood plasma metabolites have also been used to predict symptom severity in patients with clinical depression,^16^ and to differentiate between patients with depression and control individuals.^17^ Transcriptome studies have similarly revealed peripheral gene expression biomarkers with moderate predictive ability for depressive states.^18^^,^^19^ Notably, both genetic and gene-expression studies have consistently found evidence of lipid metabolism dysregulation associated with depressive symptoms.^20^, ^21^, ^22^, ^23^ For example, a Mendelian randomization analysis of 188,577 lipid and 480,359 depression GWAS-identified traits found a causal association between triglycerides and depressive symptoms, as well as deliberate self-harm and suicidal behaviour.^24^

Consistent with the findings of genetic and gene expression studies linking lipid metabolism to depression, lipids have demonstrated an unusually strong association with psychiatric disorders among various molecular phenotypes examined. Numerous studies directly investigating the blood plasma lipidome composition in depression and other psychiatric disorders have revealed significant alterations when compared to healthy controls.^25^, ^26^, ^27^, ^28^, ^29^, ^30^, ^31^, ^32^, ^33^, ^34^, ^35^, ^36^, ^37^, ^38^, ^39^, ^40^, ^41^ Further, several of these studies proposed computational models for distinguishing depressed patients from healthy individuals based on blood lipid profiles, including a predictive model with an area under the receiver operating characteristic curve (ROC AUC) of up to 0.87 in validation analysis, an accuracy that studies of other types of molecular biomarker have not achieved.^29^ In our prior multi-cohort analysis of blood lipidome alterations in schizophrenia, bipolar disorder, and major depressive disorder, we similarly developed a model that differentiated psychiatric patients from controls with high accuracy (ROC AUC = 0.86–0.95).^42^ These outcomes indicate that lipids show promise as potential biochemical markers of psychiatric disorders, including depression.

The transition from observed molecular differences between healthy individuals and patients with psychiatric conditions to a clinically applicable test for psychiatric risk assessment necessitates extensive validation and substantial effort. As an initial step towards this objective, our study explored the potential of using blood lipid profiles as a screening tool in the general population with an aim of detecting individuals exhibiting symptoms of anxiety or depression. We assessed blood plasma lipid levels in 604 volunteers using a high-throughput and reliable lipidome analysis method with prospective clinical applicability, direct-infusion mass spectrometry. These volunteers were sampled from the population of Moscow, Russia, and their lipid levels were investigated for any association with self-reported symptoms of anxiety and depression. To further substantiate our findings, we compared these associations with alterations observed in 32 patients with a clinical depression diagnosis. Accordingly, we investigated the possibility of generalizing lipid alterations from a clinical cohort to the detection of individuals with self-reported symptoms of mental disorders.

## Methods

### Study participants

Patients with a diagnosis of major depressive disorder (*n* = 32; mean age ± std = 33 ± 13; 47% female) were recruited from the Mental Health Clinic no. 1 named after N.A. Alexeev of the Department of Health of Moscow. Inclusion criteria consisted of a diagnosis of major depressive disorder established during an inpatient examination according to the International Classification of Diseases (ICD-10, code F32 or F33). Matched controls without mental disorders (*n* = 21; mean age ± std = 29 ± 8; 52% female) were recruited in parallel, including individuals without psychiatric disorder diagnosis. Volunteers (*n* = 604; mean age ± std = 30 ± 10; 72% female) were recruited from the Moscow population, and were additionally asked to complete HADS self-reporting questionnaires (Figure S1). Records with missing HADS scales values or demographic data were not included in the study. The HADS scale consists of two subscales: subscale A (assessment of anxiety) and subscale D (assessment of depression). Each subscale includes 7 statements. Each statement has 4 response options reflecting the degree of severity of the symptom and is coded in increasing levels of symptom severity from 0 (absence) to 3 (maximum severity). The scale was validated for the Russian population.^43^ The following ranges of HADS-A/D scores were considered: healthy ranges HADS-A/D ≤ 7, mild symptoms of anxiety/depression 8 ≤ HADS-A/D ≤ 10, moderate symptoms 11 ≤ HADS-A/D ≤ 14, severe symptoms HADS-A/D ≥ 15.

Volunteer participants were selected according to consecutive sampling scheme: the study sample was formed in 2023 from the 747 volunteers who responded to a social media announcement. The announcement included information about the study, location of the study, inclusion and exclusion criteria. A number of volunteers filled out questionnaires but failed to show up for blood collection, and were not included in the study, giving a final total of blood samples with lipidome measurements from 604 individuals (Figure S1). Volunteer sample size was chosen so that the expected number of individuals with depression-like symptoms would be >30, matching clinical depression sample size, assuming an approximate 5% incidence of depressive symptoms in the population.^1^, ^2^, ^3^ Exclusion criteria for all groups were age (<18 or >70 years old), substance abuse, intellectual disability, severe somatic or neurological diseases that may affect a diagnosis of mental disorder, in line with ICD diagnostic criteria.

### Ethics

Informed consent was obtained from all participants. The study was conducted according to the guidelines of the Declaration of Helsinki formulating ethical principles for medical research involving human subjects. The protocol of this study was reviewed and approved by local ethical committee (Protocol No.2/25.01.2022).

### Plasma collection

Plasma was obtained from peripheral venous blood in the morning. Plasma samples were collected in 4 ml Vacutainer tubes containing the chelating agent ethylenediaminetetraacetic acid (EDTA) (Vacuette, Greiner bio-one, Austria). Tubes were centrifuged at room temperature at 1500*g* for 15 min. The supernatant was stored in 500 μl aliquots at −80 °C.

### Lipid extraction

Plasma samples were randomized before the extraction. Extraction blanks were added in the end of the extraction batch and represented empty samples. An aliquot of 250 μl of water was added to 20 μl of plasma, following with the addition of 1300 μl of cold mixture MTBE:MeOH (7:2, v:v). After 10 min of ultrasound exposure (50/60 Hz, Bandelin Electronic, Berlin, Germany) in ice bath, samples were vigorously shaken for 40 munities at 4 °C (Vortex Genie, Scientific Industries, New York, USA) and centrifuged afterwards for 10 min at 12,700 rpm, 4 °C (Centrifuge 5427R, Eppendorf, Germany). Then 1000 μl of upper phase was collected to a separate tube and dried under reduced pressure (20Pa) at 30 °C (Concentrator plus, Eppendorf, Germany). Dried pellets were stored until the analysis at −80 °C. On the day of mass spectrometry measurements, pellets were reconstituted in 200 μl of mixture of isopropanol:methanol:chloroform in a mixture of (4:2:1; v:v:v) and diluted 5-fold with the mixture of isopropanol:methanol:chloroform in a mixture of (4:2:1; v:v:v) using 9.5 mM ammonium formate as additive.

### Mass spectrometry data acquisition

Mass spectrometry analysis was performed in positive mode using a QExactive mass spectrometer (Thermo Fisher Scientific, USA) equipped with a heated electrospray ionization source. Samples were introduced by a flow injection using a Waters Acquity UPLC chromatograph (Waters, Manchester, UK). The mobile phase consisted of a 7.5 mM ammonium formate solution in isopropanol:methanol:chloroform in a mixture of (4:2:1; v:v:v). The flow rate was modulated during the analytical run. The eluent flow rate was set to 0.8 ml/min in the intervals of 0–0.04 min and 2.01–3.0 min and maintained at 10 μl/min in the range of 0.04–2.01. Increased flow rate was used for sample introduction in the beginning and loop flushing at the end of the run. One run duration time was 3 min. Injection volume was set to 20 μl, the autosampler temperature was maintained at 4 °C.

The source settings were established as follows for 10 μl/minflow: sheath gas, 15 a.u.; aux gas, 5 a.u.; sweep gas, 0 a.u.; spray voltage, 3.5 kV; capillary temperature, 250 °C; S-lens RF level, 50; aux heater temperature, 250 °C. For 800 μl/min, source parameters changed accordingly: sheath gas, 60 a.u.; aux gas, 20 a.u.; sweep gas, 4 a.u.; spray voltage, 2.5 kV; capillary temperature, 300 °C; S-lens RF level, 50; aux heater temperature, 300 °C.

Each mass spectrometry experiment consisted of full scan events and subsequent data-independent fragmentation (DIA). First, full scan range of interest (*m*/*z* 200–1050) were split into narrow windows to avoid C-trap overload. The ion acquisition program was set as follows: 0.12–0.17s: 200–652 Da, 0.17–0.22s: 652–684 Da, 0.22–0.27s: 684–716 Da, 0.27–0.32s: 716–764 Da, 0.32–0.37 s: 764–812Da, 0.37–0.42s: 812–876 Da, 0.42–0.47s: 876–908 Da, 0.47–0.52s: 908–1051 Da. Splitting intervals were selected based on spectra ion population. DIA event consisted of 1 Da-width windows within the range 200.5–1050.5 *m*/*z* with a resolution of 17,500 (FWHM at *m*/*z* 200). Collision energy was applied in a stepwise manner (15-20-25), AGC target was set at 2∗10E5, isolation window was 1.2 Da and fixed first mass equalled to *m*/*z* 80. All spectra were recorded in profile mode. Pierce LTQ Velos ESI Positive Ion Calibration Solution was used for external calibration in positive mode. Resolution was set at 140,000 (FWFM at *m*/*z* 200) with an AGC setting of 5∗10E6 and max IT time 100 ms.

Quality control samples (QC) were made from pooled aliquots of first 96 samples in the batch. QC samples were inserted after every 12 samples to account for batch effects and technical reproducibility and in the beginning of each batch to allow for system equilibration. Long-term-reference (LTR) samples were inserted every 12 samples, as well, to account for possible intensity drifts between experiments.

### Data pre-processing and lipid identification

Raw files were converted to.mzXML format with PeakStrainer software keeping only MS1 information for biological samples and extraction blanks (time range of 0–32.5 s) and MS1 and MS2 information for QC samples. Then.mzXML files were loaded to LipidXplorer software (v. 1.2.8.1),^44^ using the import settings taken from^45^ with MS2 threshold of 20,000 abs. Lipid identification were based on MFQL (molecular fragmentation query language) scripts downloaded from article,^45^ along with customized MFQL files for isotopically labelled standards. The following lipid classes were included into analysis CAR, LPC, LPC O-, LPE, SM, TAG, DAG, PC, PC O-, PE, PE P-, PI, CE, Cer. Lipid identification strategy was based on precursor high resolution MS1 information and MS2 fragmentation data.

### Data post-processing

Features with more than 10% of zero values across plasma samples were removed from the analysis. The remaining zero values were replaced by 0.9 of the minimum non-zero value across plasma samples in each feature. Feature intensities were transformed with base-2 logarithm (log2). Contaminants were removed using extraction blank samples according to the following rule: mean log2 intensity of plasma samples − mean log2 intensity of blank samples <1. For each feature, measurement batch effect (consisting of 96 plasma samples each) was corrected by subtracting the median intensity of QC samples in this experimental batch. The feature intensities were then returned to their original scale by adding the corresponding median value across all batches. Features retaining high technical variability after batch correction were removed using QC samples according to the following rule: features with standard deviation across QC samples >0.5 (in log2 scale) were removed from the analysis. Plasma samples measurements were conducted in two large temporal batches, and LTR samples were used to align intensities of the two batches. For each feature, the difference of median log2 intensity values in LTR samples between the second and first experimental batches was added to the log2 intensity value of the second experimental batch.

### Statistics

Python version 3.7.3 was used for statistical analysis.

To adjust lipid abundances for age, sex, and BMI prior to conducting the association analysis with the HADS scales, we used a linear model regressing age, sex, and BMI on each lipid feature (Python package sklearn.linear_model.LinearRegression). Corresponding residual values were used for Pearson correlation analysis with HADS-A/D values (scipy.stats.pearsonr).

To investigate whether there was a significant association between corrected blood plasma lipid levels and the HADS-A/HASD-D scores, we performed a permutation test, by randomly shuffling the HADS-A/HASD-D scores across individuals and calculating the number of lipids with a Benjamini-Hochberg corrected *p*-value less than 0.1. The permutation *p*-value was calculated as the proportion of permutations, from 1000, for which this number was equal to or larger than the same number calculated for the original data.

Hypergeometric test (one-sided Fisher test) was used to test for overrepresentation of lipid classes among significant lipids (scipy.stats.hypergeom). Enrichment ratio for over-representation analysis of ether phospholipids among the eight lipids displaying the strongest associations with the HADS-D scale (significant lipids) was calculated by dividing the number of lipids from the particular biochemical group among the significant lipids by the corresponding expected number, estimated as N_sign_ × $\frac{N_{group}}{N_{total}}$ (where N_sign_ is the number of significant lipids, N_group_ is the total number of lipids from the particular biochemical group, and N_total_ is the total number of lipids). Double bond index for PUFA over-representation analysis was calculated as the total number of double bonds divided by the number *k* of side chains in the lipid structure (*k* = 3 for TAG, *k* = 1 for lyso-species, CE, and CAR, and *k* = 2 for the rest of the lipid classes).

Mann–Whitney U test was used to test whether significant lipids showed a difference in the number of double bonds compared to the rest of the lipids (scipy.stats.mannwhitneyu).

Python package sklearn.linear_model LogisticRegression with penalty = ‘l1′ was used for predictive modelling. Of note, the regularization parameter C is sensitive to training sample size, hence the number of features chosen by the lasso logistic model was considered when choosing the appropriate parameter value. First, model performance was estimated for different parameters: C = 0.01, 0.1, 0.5, 10, 100, 500 1000 (Figure S2) in randomized cross validation: 1000 random test-train splits were preformed, for which *k* = 10 control and *k* = 10 disease samples were chosen at random from the *n* = 68 samples (Table S1), and the rest (*k* = 48) were used for training a lasso logistic regression model. The data was normalized by the mean and standard deviation for each feature across the 68 samples. We chose C = 0.5 (Figure S2), corresponding to 14.9 ± 2.4 predictor on average (mean ± std), for reporting model performance in separating healthy controls from depression patients using randomized cross-validation (same train-test split approach described above). For predicting risk scores of volunteers, we used all samples *n* = 68 in model training, with parameter C = 0.3, corresponding to 14 predictors chosen by the model (Table S2). The data was also normalized by the mean and standard deviation for each feature across the 68 samples. Standard cutoff of 0.5 of predicted scores was used for defining positive and negative classes. For subsequent correlation assessment between predicted risk scores and HADS scales (Spearman correlation for all values; Pearson correlation for averaged prediction scores across discrete HADS-A/D values; scipy.stats.spearmanr and scipy.stats.pearsonr), as well as ROC AUC value estimation for the detection of volunteers with increased HADS-D scores, the *n* = 15 volunteers used in model training were excluded from the analysis.

The 95% confidence intervals for correlation coefficients were estimated using 10,000 bootstrap resampling and calculating the (2.5%, 97.5%) quantile values. For predictive modelling performance estimation, we report 95% subsampling interval by calculating the (2.5%, 97.5%) quantile values for the performance values in test subsamples in the train/test splitting used during randomized cross-validation.

### Role of funders

The funders had a supporting role in data collection, and no role in the study design, data analyses, interpretation, or writing of report.

## Results

### Cohort description

**Fig. 1:**
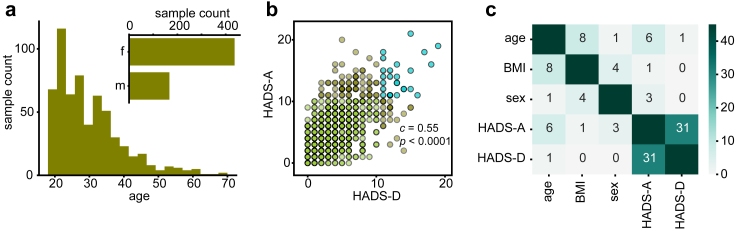
Demographic and mental health characteristics of the volunteer cohort (*n* = 604). (a) The age and sex distribution among the volunteers in the cohort. (b) The distribution of self-reported anxiety (HADS-A) and depression (HADS-D) symptom scores among the individuals in the cohort. Colours indicate severity of symptoms: green represents no or mild symptoms (0–10) on both scales, brown represent moderate or severe symptoms (≥11) on one of the scales, and blue indicates moderate or severe symptoms (≥11) on both scales. Colour intensity is proportional to the number of individuals having the respective scores. Pearson correlation coefficient and *p*-value is indicated on the plot. (c) The co-dependency among demographic factors and self-reported clinical indicators. The numbers depicted represent the percentage of variation (R^2^) explained for a specific variable by another variable, as determined by linear regression model analysis.

То evaluate the potential of using blood lipid profiles as indicators of mental disorders in the general population, we conducted a study of 604 urban population representatives with exclusion criteria limited to age (>70 years) and severe and decompensated somatic or neurological diseases (age 29.9 ± 9.6 years; 72% female; Fig. 1a; Table S3). For each of the volunteers we collected blood plasma samples and anxiety and depression scores assessed using the Hospital Anxiety and Depression Scale (HADS) questionnaire for symptom assessment, with the HADS-A and HADS-D subscales providing the anxiety and depression scores, respectively.^46^

As anticipated from a cohort representative of the general population, most individuals scored within the healthy range for both scales (65% and 81% respectively for HADS-A and HADS-D ≤7). A minor percentage reported severe (3% and 1.5% respectively for HADS-A/D ≥ 15) and moderate (12% and 6.5% for 11 ≤ HADS-A/D ≤ 14) symptoms of depression or anxiety, with the rest of the cases classified as having mild symptoms (8 ≤ HADS-A/D ≤ 10). Consistent with prior observations, the HADS-A and HADS-D scores were significantly correlated with each other (Pearson correlation *c* = 0.55, *p* < 0.0001, 95% CI = (0.49, 0.61); Fig. 1b, Figure S3).^46^ Further, among three examined demographic factors—age, sex, and BMI—HADS-A scores demonstrated weak yet significant correlations with age and sex (linear regression, *p* < 0.0001, R^2^ = 6% and 3%, respectively; Fig. 1c, Figure S3; Table S4). The HADS-D scores showed an even weaker association with age and none with sex or BMI (*p* = 0.010, R^2^ = 1% for age; Fig. 1c, Figures S3 and S4; Table S4).

### Lipid associations

**Fig. 2:**
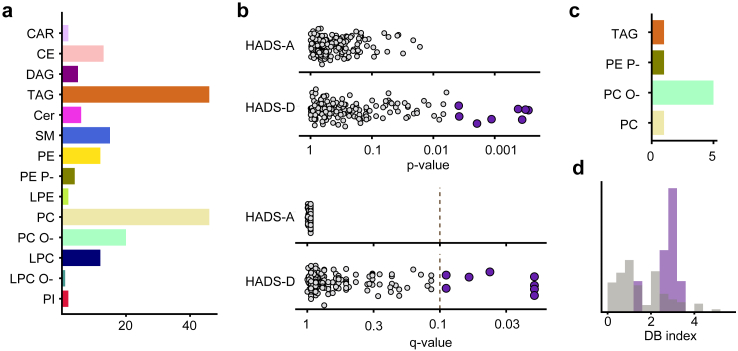
Lipidome associations with HADS-A and HADS-D scores. (a) The measured lipid classes and the number of species in each one. CAR indicates acylcarnitine; CE, cholesteryl ester; DAG, diacylglycerol; TAG, triglyceride; Cer, ceramide; SM, sphingomyelin; PE, phosphatidylethanolamine; PE P-, plasmanyl/plasmenyl phosphatidylethanolamine; LPE, lysophosphatidylethanolamine; PC, phosphatidylcholine; PC O-, plasmanyl/plasmenyl phosphatidylcholine; LPC, lysophosphatidylcholine; LPC-O, lyso plasmanyl/plasmenyl phosphatidylcholine; PI, phosphatidylinositol. (b) *p*-value (top) and q-value (FDR-corrected *p*-value, bottom) distributions for the Pearson correlation analysis between abundances of lipids and HADS-A or HADS-D scores (*n* = 604). Dotted line separates 10% FDR threshold, and the eight lipids significantly associated with HADS-D are coloured in dark blue. (c) For lipids significantly associated with HADS-D scores, the distribution by lipid classes. (d) For lipids significantly associated with HADS-D scores, the double bond index distribution (purple) compared to other lipids (grey). Double bond index was defined as the number of double bonds divided by the number of side chains in the lipid structure.

We then moved to examining the association between blood plasma lipid levels and self-reported anxiety and depression scores. Direct-infusion mass spectrometry measurements of 604 blood plasma samples, followed by data processing and quality control filtration, yielded the abundance levels for 186 lipids representing 14 chemical classes (Fig. 2a; Table S5). Recognizing that demographic factors can have a substantial impact on plasma lipid levels,^47^ we adjusted the lipid abundances for age, sex, and BMI prior to conducting the association analysis with the HADS scales. A significant correlation with corrected blood plasma lipid levels was found for the HADS-D scale (Pearson correlation, permutation test *p* = 0.026; Fig. 2b; Table S5), whereas no statistically significant association was detected for the HADS-A scale (Pearson correlation, permutation test *p* = 1; Fig. 2b; Table S5).

Among eight lipids that displayed the strongest associations with the HADS-D scale (PC 38:6, PC O-34:3, PC O-36:5, PC O-36:6, PC O-38:6, PC O-38:7, PE P-38:6, TAG 58:8; Pearson correlation, FDR = 10%; Table 1, Table S6), five represented the PC O- lipid class and, more generally, six represented ether lipids. This prevalence of ether lipids was far above the chance expectation indicating biochemical specificity of the altered compounds (hypergeometric test, one-sided Fisher test, enrichment ratio = 5.8, *p* = 0.00040 for PC O-, enrichment ratio = 5.8, *p* < 0.0001 for PC O- and PE P- merged; Fig. 2c). All eight lipids also displayed significantly higher polyunsaturated fatty acids (PUFAs) incidence in their structure, with the median of double bond per side chain equalling 3.0 compared to 1.3 for the remaining quantified lipids (Mann–Whitney U test, *p* = 0.0014; Fig. 2d).

**Table 1:** Results of correlation analysis between lipid abundance levels and HADS-D scores (*n* = 604).

Lipid	Lipid class	c	95% CI	*p*-value	q-value
PC O-34:3	ether phosphatidylcholine	−0.15	(−0.23, −0.06)	0.00028	0.019
PC O-36:6	ether phosphatidylcholine	−0.15	(−0.22, −0.07)	0.00032	0.019
PC O-38:7	ether phosphatidylcholine	−0.14	(−0.22, −0.06)	0.00036	0.019
PE P-38:6	ether phosphatidylethanolamine	−0.14	(−0.22, −0.06)	0.00042	0.019
PC O-38:6	ether phosphatidylcholine	−0.13	(−0.21, −0.05)	0.0011	0.042
PC O-36:5	ether phosphatidylcholine	−0.13	(−0.21, −0.04)	0.0019	0.060
PC 38:6	phosphatidylcholine	−0.12	(−0.19, −0.04)	0.0038	0.090
TAG 58:8	triglyceride	−0.12	(−0.20, −0.04)	0.0039	0.090

The table lists lipids with the strongest associations, the corresponding Pearson correlation coefficients (“c”), 95% confidence intervals (CI) for the correlation coefficients, *p*-values, and FDR-corrected *p*-values (“q-value”).

**Fig. 3:**
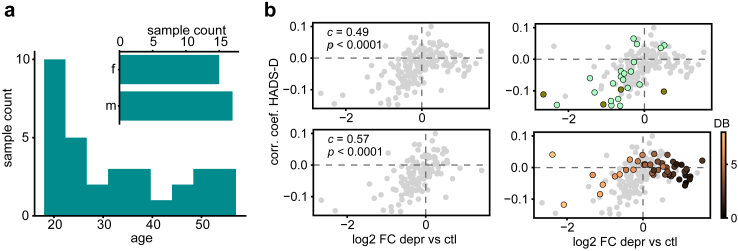
Congruence of lipidome alterations in volunteer cohort and clinical depression patients. (a) Age and sex distribution for the dataset of patients with clinical depression (*n* = 32). (b) Relationship between the association of HADS-D and lipid abundances (Pearson correlation coefficients) and alterations in lipid abundances in clinical depression (mean base-2 log transformed fold-changes between clinical depression and the volunteer cohort). Spearman correlation coefficient and *p*-value of the relationship is indicated on the corresponding plots (*n* = 186 lipids). Top left: all lipids; bottom left: all lipids without triglycerides; top right: ether phospholipids are highlighted in colour (PC O-, PE P-, LPC O-, shades correspond to Fig. 2a); bottom right: triglycerides are highlighted in colour, the shades indicate the total number of double bonds.

Several studies have reported substantial alterations in blood lipidome profiles of patients with clinical depression.^27^, ^28^, ^29^, ^30^, ^31^, ^32^^,^^41^ If the results we found for the volunteer cohort reflect depression-associated metabolic alteration, they would presumably align with those observed in patients with major depression diagnosis. To assess the validity of this notion, we collected blood samples and measured blood plasma lipid levels in 32 psychiatric inpatients diagnosed with clinical depression, using the same procedure as for the volunteer cohort (Fig. 3a; Table S7). The alterations found in these psychiatric patients were consistent with the results of the HADS-D analysis. Specifically, the alterations in lipid abundances of depression patients compared to the volunteer cohort, which represented the general population, were congruent with the associations between lipids and HADS-D scores identified using the volunteer cohort alone (Spearman correlation, *c* = 0.49, *p* < 0.0001, 95% CI = (0.36, 0.60); Fig. 3b). Further, when considering only ether-phospholipid classes, PC-O and PE-P, which showed the strongest association with depression symptoms for volunteers, the correlation increased (Spearman correlation, *c* = 0.67 *p* = 0.00035, 95% CI = (0.35, 0.85); Fig. 3b). The congruence of these lipid alterations between the two analyses is further supported by consistent reports of decreased ether phospholipids in the blood of depressed individuals.^28^, ^29^, ^30^

While lipids showed parallel alterations in high HADS-D volunteers and patients with clinical depression, one class, triglycerides, substantially contributed to the discrepancy between these two groups (excluding triglycerides, Spearman correlation, *c* = 0.57, *p* < 0.0001, 95% CI = (0.43, 0.68); Fig. 3b). Among triglycerides, we noted a clear relationship between the occurrence of double bonds in the fatty acid residues and the extent of disagreement. Specifically, the most saturated and shorter-chained triglycerides showed the greatest discrepancy, while triglycerides containing polyunsaturated fatty acids (PUFAs) followed the overall positive correlation trend (Fig. 3b, Figure S5).

### Predictive modelling

**Fig. 4:**
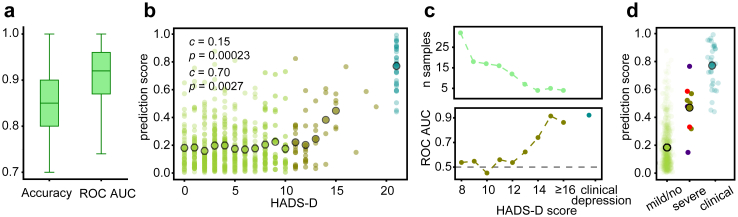
Predictive modelling for the detection of individuals with high HADS-D scores. (a) Boxplots illustrating the randomized cross-validation accuracy and ROC AUC values of the model separating clinical depression from healthy controls (*n* = 32 and *n* = 36, respectively). Standard boxplot definition was used for illustration (the box extends from the first quartile to the third quartile of the data, with a line at the median; the whiskers extend from the box to the farthest data point lying within 1.5x of the inter-quartile range from the box). (b) The relationship between volunteers' HADS-D values and their predicted scores derived from the model trained on clinical depression patients versus controls (*c* and *p* on the top: Spearman correlation coefficients and *p*-values, *n* = 589, excluding 15 volunteer individuals used in model training). Individual predictions are represented by coloured points, with mean predicted scores for each HADS-D value represented by larger circles (*c* and *p* below: Pearson correlation coefficients and *p*-values for averaged prediction scores across discrete HADS-D values, *n* = 16). Light green illustrates volunteers with no or mild symptoms (HADS-D = 0–10), dark green illustrates those with moderate to severe symptoms (HADS-D ≥ 11), and teal blue symbols on the right illustrate clinical depression patients. (c) Top: the number of individuals with specific HADS-D scores. Bottom: ROC AUC values of the model performance in distinguishing individuals with the specific HADS-D scores (dark olive), as well as clinical depression (teal blue), from those without depressive symptoms (HADS-D ≤ 7). (d) Depression probability scores generated by the model for volunteers with no or mild symptoms (HADS-D ≤ 10), those with severe self-reported depression (HADS-D ≥ 15), and hospitalized patients with clinical depression. Individual predictions are represented by coloured points, while the predicted scores averaged in each group are illustrated by larger outlined circles. For the group of volunteers with severe depressive symptom, red indicates those on prescribed antidepressants, blue indicates non-medicated individuals, and olive represents those with unconfirmed medication status.

After observing the alignment of blood plasma lipidome alterations in individuals with clinical depression and the volunteers with elevated depression scores, we considered training a mathematical model to separate clinical depression from healthy individuals with the aim to extend its predictions to the volunteer individuals. Accordingly, we first used lipid measurements for 32 patients with depression and 36 individuals with no signs of mental illness (*n* = 21 matched controls, *n* = 15 individuals from the volunteer cohort with HADS-D and HADS-A ≤ 7; Table S8) to train a lasso logistic regression predictive model (Table S2). In randomized cross-validation, the model successfully differentiated the two groups: patients with clinical depression and controls showing no depression symptoms (mean ROC AUC = 0.91, 95% subsampling interval = (0.77, 1); mean accuracy = 0.83, 95% subsampling interval = (0.70, 95); mean sensitivity = 0.89, 95% subsampling interval = (0.70, 1); mean specificity = 0.77, 95% subsampling interval = (0.60, 1); Fig. 4a, Figure S6).

Using this model trained to separate patients with clinical depression from healthy controls, we then calculated the depression probability scores for the volunteer cohort. In agreement with our predictions, depression probability scores calculated by the model correlated positively and significantly with volunteers’ HADS-D scores (Spearman correlation *c* = 0.15, *p* = 0.00023, 95% CI = (0.06, 0.24) for all values; Pearson correlation *c* = 0.70, *p* = 0.0027, 95% CI = (0.51, 0.85) for averaged prediction scores across discrete HADS-D values; Fig. 4b, Figure S7). Further, despite substantial correlation between HADS-D and HADS-A scales, the association between the predictive model scores and HADS-A values was notably weaker, suggesting a degree of specificity of the model in detecting depression-associated alterations (Spearman correlation *c* = 0.10, *p* = 0.021, 95% CI = (0.01, 0.18) for all values; Pearson correlation *c* = 0.47, *p* = 0.041, 95% CI = (−0.01, 0.77) for averaged prediction scores across discrete HADS-A values; Figure S8).

The ability of the model to distinguish between individuals without depressive symptoms (HADS-D ≤ 7) and those displaying moderate signs of depression, although consistently better than expected by chance, improved substantially with greater HADS-D threshold defining the risk group. Specifically, model ROC AUC values increased from 0.64 to 0.84 for individuals selected using HADS-D ≥ 11 (*n* = 48) and HADS-D ≥ 14 (*n* = 13), respectively (Fig. 4c, Figure S9). For volunteers with severe signs of depression (HADS-D ≥ 15, *n* = 9), the accuracy of their differentiation from control individuals was similar to that of individuals with clinical depression (ROC AUC = 0.89 and 0.92 for depressed volunteers and clinical depression, respectively; Fig. 4c, Figure S9).

Previous studies indicated potential influence of antidepressant medication on the blood plasma lipid composition.^48^, ^49^, ^50^ Given that undisclosed medication use could confound results for volunteers with depressive symptoms, the nine volunteers with severe signs of depression were invited for a follow-up visit. Of the five individuals who responded, two were currently receiving treatment for depressive symptoms, while three were medication-naïve (Table S3). There were no observable differences in the model prediction score distributions between medicated and medication-naive individuals (Fig. 4d). Although based on a small sample size, this observation nonetheless indicates lack of association between model prediction scores and medication status.

## Discussion

Our results demonstrate a consistent and reliable association between blood plasma lipid levels and self-reported mental health symptoms within a cohort of volunteers representing the general urban population. Despite a significant overlap between self-reported depression and anxiety symptoms, assessed using HADS-D and HADS-A scores, the observed lipidome alterations showed a degree of specificity to manifestations of depression. This relationship was further supported by the congruence of lipidome alterations found in volunteers and those found in the blood plasma of patients diagnosed with major depressive disorder.

Biochemically, lipids associated with the severity of depressive symptoms in the volunteer cohort were predominantly found in specific groups, namely ether phospholipids and lipids containing polyunsaturated fatty acids, both of which have been previously linked to depression. For example, a decrease in ether phospholipids in the blood has been noted in a family-based depression study and an investigation of HADS-D associations including both healthy and depressed individuals.^30^^,^^51^ Similarly, there is strong evidence for polyunsaturated fatty acids deficiency being related to depression.^52^, ^53^, ^54^, ^55^, ^56^, ^57^

Our results strongly suggest congruent lipidome alterations in the blood plasma of both psychiatric patients diagnosed with clinical depression and individuals with depressive symptoms within a general population cohort. Notably, more saturated, shorter-chained triglycerides were the main biochemical group to display a distinctly inconsistent alteration profile between volunteers and patients with depression. One possible explanation for this inconsistency could be related to differences in metabolic health, considering that this particular triglyceride signature has been shown to be predictive of insulin resistance, diabetes, and non-alcoholic fatty liver disease, and possibly even superior in this regard to standard lipid profiling.^58^^,^^59^ Such differences between patients with depression and volunteers would be in line with expected increased instances of antidepressant usage and related metabolic changes among patients with psychiatric conditions.^60^ Alternatively, it is intriguing to speculate that these triglyceride alterations might reflect the symptom severity distinction between functioning volunteers with high depression scores and individuals hospitalized for their depressive state. Lending support to this hypothesis, we have previously shown that the same triglyceride signature was associated with impaired treatment response in schizophrenia patients.^61^

The general agreement between alterations in the blood plasma lipidome observed in clinical depression patients and volunteers with higher depression scores suggests the potential of using a predictive model based on psychiatric patients' lipid levels data as a screening tool for depression. Prior studies have demonstrated the feasibility of developing predictive models that could effectively distinguish patients diagnosed with major depressive disorder from healthy controls.^28^^,^^29^^,^^32^ Likewise, in our analysis, we successfully developed a predictive model that differentiated between patients with clinical depression and healthy individuals based on the abundance levels of specific blood plasma lipids (ROC AUC = 0.91, 95% subsampling interval = (0.84, 1)). Despite the small number of individuals used in the model training (*n* = 32 and 36), its application to the lipidome data from the volunteer cohort revealed a significant positive correlation between the model's depression probability scores and HADS-D values. This outcome demonstrates both the robust association of observed lipidome alterations with depressive states, specifically, and the possibility of generalizing lipid alterations from a clinical cohort to the general population. The relatively low correlation strength in the association analysis between model prediction scores and the HADS-D scale resulted from the inherent imbalance in the dataset's symptom severity distribution. Most volunteers (81%) reported no signs of depression (0–7 scores of HADS-D), while only 6.5% and 1.5% stated moderate or severe symptoms of depression, respectively. Therefore, applying the model to individuals within increasingly restricted HADS-D score brackets substantially enhanced model accuracy, ultimately distinguishing volunteers with the most severe depressive symptoms from healthy individuals with reliability comparable to that achieved for clinical depression patients (ROC AUC = 0.89–0.92). Although this result requires verification by further studies involving larger cohort sizes, it nonetheless suggests that our approach could lead to accurate detection of individuals afflicted with depressive conditions based on their blood lipid level profiles.

Our study has several limitations. One such limitation is the restricted range of blood plasma lipids that we were able to assess due to our reliance on a direct-infusion mass spectrometry protocol. Although this method facilitates the rapid screening of large sample cohorts, it compromises sensitivity, particularly for specific lipid classes like ceramides, which have been reported to be consistently associated with depression levels.^62^ Another limitation is the relatively small number of individuals diagnosed with clinical depression used for the predictive model training, as well as the low number of volunteers with high HADS-D scores that were needed for model testing. While the congruence between lipidome alterations we find in our study for clinically diagnosed depression patients and volunteers with elevated HADS-D scores partially mitigates this concern, since the likelihood of such congruence is low regardless of sample size, caution should nevertheless be exerted with the generalization of statistical modelling due to possible model misspecification, as well as unmeasured confounding factors, such as medication use. One further limitation is the reliance of our model's performance measure in the volunteer cohort on self-reported HADS-D scores, and not a clinical assessment of depressive symptoms. The correlation between clinician-rated severity of depressive symptoms and HADS-D scores has been shown to be limited.^46^ Moreover, the high HADS-D group may encompass a diverse population in terms of diagnoses, potentially including not only individuals suffering from affective mental diseases, but subjects exhibiting post-traumatic stress disorder (PTSD) symptoms or those afflicted with psychotic disorders. Of note, our model demonstrated a certain degree of specificity to the detection of individuals with elevated depression scores, in particular, despite the well-documented association between HADS-D and HADS-A scales.^46^ Nevertheless, it is difficult to presume the specificity of the observed lipid alterations to any one mental disorder without additional evidence. Likewise, due to the cross-sectional design of the study, only symptoms at baseline were collected, which bars the evaluation of the lipid-based risk scores as potential prognostic markers. These latter two points would constitute main study objectives for future investigations.

In conclusion, despite certain limitations, our study utilizing a high-throughput method for blood lipidome measurements has revealed a significant association between lipid abundances and self-reported depressive symptoms. We have presented a lipid-based model that shows promising reliability in identifying individuals from the general population with severe self-reported depressive symptoms. Although the precise implications of these findings are currently challenging to predict, they undeniably highlight the potential utility of lipid-based panels in detecting individuals at heightened risk of psychiatric disorders.

## Contributors

GK and PK—conceptualization, supervision, writing review & editing. AM, YZ, DA, DR, EG, AO, and VS—investigation (data collection), methodology. ES—methodology, validation, investigation (experiments), writing review & editing. AGol and AS—investigation (experiments). AGon, DP, IA—project administration, resources. AT—formal analysis, visualization, writing original draft, writing review & editing. AT and ES have accessed and verified the data, PK was responsible for the decision to submit the manuscript. All authors have read and approved the final version of the manuscript.

## Data sharing statement

Lipid data used in statistical model construction, including normalized log2 lipid abundances for patients with depression and controls, is available as a supplementary tables (S1, S8).

## Declaration of interests

The authors declare no conflict of interest.

## Appendix A Supplementary data

Supplementary Figures

Supplementary Tables **Table S1.** Normalized lipid abundances for patients with clinical depression and controls used in the model training. For each feature, log2 lipid abundances were normalized by mean and standard deviation. **Table S2.** Predictors selected by the lasso logistic regression model. **Table S3.** Volunteer cohort demographic and HADS questionnaire data, as well as model prediction scores. **Table S4**. P-values of linear regression between demographic and HADS questionnaire variables. **Table S5.** Quantified lipids and their correlations with HADS scores. **Table S6.** Results of correlation analysis between lipid abundance levels and HADS-D scores, calculated separately for females and males. **Table S7.** Depression cohort demographic data. **Table S8.** Non-depression samples used in predictive model training.
