# Supplementary material for: Screening for depression in the general population through lipid biomarkers
Source: eBioMedicine. 2024 Nov 20;110:105455. doi: 10.1016/j.ebiom.2024.105455 (PMC11617895; doi:10.1016/j.ebiom.2024.105455)
Supplement: Supplementary Figures [file mmc1.docx]

**Screening for Depression in the General Population through Lipid Biomarkers**

Anna Tkachev^1,2§^, Elena Stekolshchikova^1,§^, Anastasia Golubova^1^, Anna Serkina^1^, Anna Morozova^3,4^ , Yana Zorkina^3,4^, Daria Riabinina^3^, Elizaveta Golubeva^3^, Aleksandra Ochneva^3^, Valeria Savenkova^3^, Daria Petrova^1^, Denis Andreyuk^3,5^, Anna Goncharova^6^, Irina Alekseenko^7^, Georgiy Kostyuk^3,*^, Philipp Khaitovich^1,2*^

^1^Vladimir Zelman Center for Neurobiology and Brain Rehabilitation, Skolkovo Institute of Science and Technology, Moscow, 121205, Russia

^2^LLC NeurOmix, Moscow, 119571, Russia

^3^Mental-health Clinic No. 1, named after N.A. Alekseev, Moscow, 117152, Russia

^4^Department of Basic and Applied Neurobiology, V. Serbsky Federal Medical Research Centre of Psychiatry and Narcology, 119034 Moscow, Russia.

^5^Economy Faculty, M.V. Lomonosov Moscow State University, 119991 Moscow, Russia

^6^Moscow Center for Healthcare Innovations, Moscow, 123473, Russia

^7^Shemyakin-Ovchinnikov Institute of Bioorganic Chemistry, Russian Academy of Science, Moscow region, 142290, Russia

^§^ contributed equally

^*^ corresponding authors

Georgiy Kostyuk: kgp@yandex.ru

Philipp Khaitovich: khaitovich@gmail.com

**Supplementary Figures.**

Questionnaires received – 747

Blood samples collected – 620

People matching the inclusion/ exclusion criteria – 743

Blood samples with lipidome measurements – 604

Complete questionnaires without errors or missing data – 604

**Figure S1.** Flow chart of volunteer samples recruited for the study.


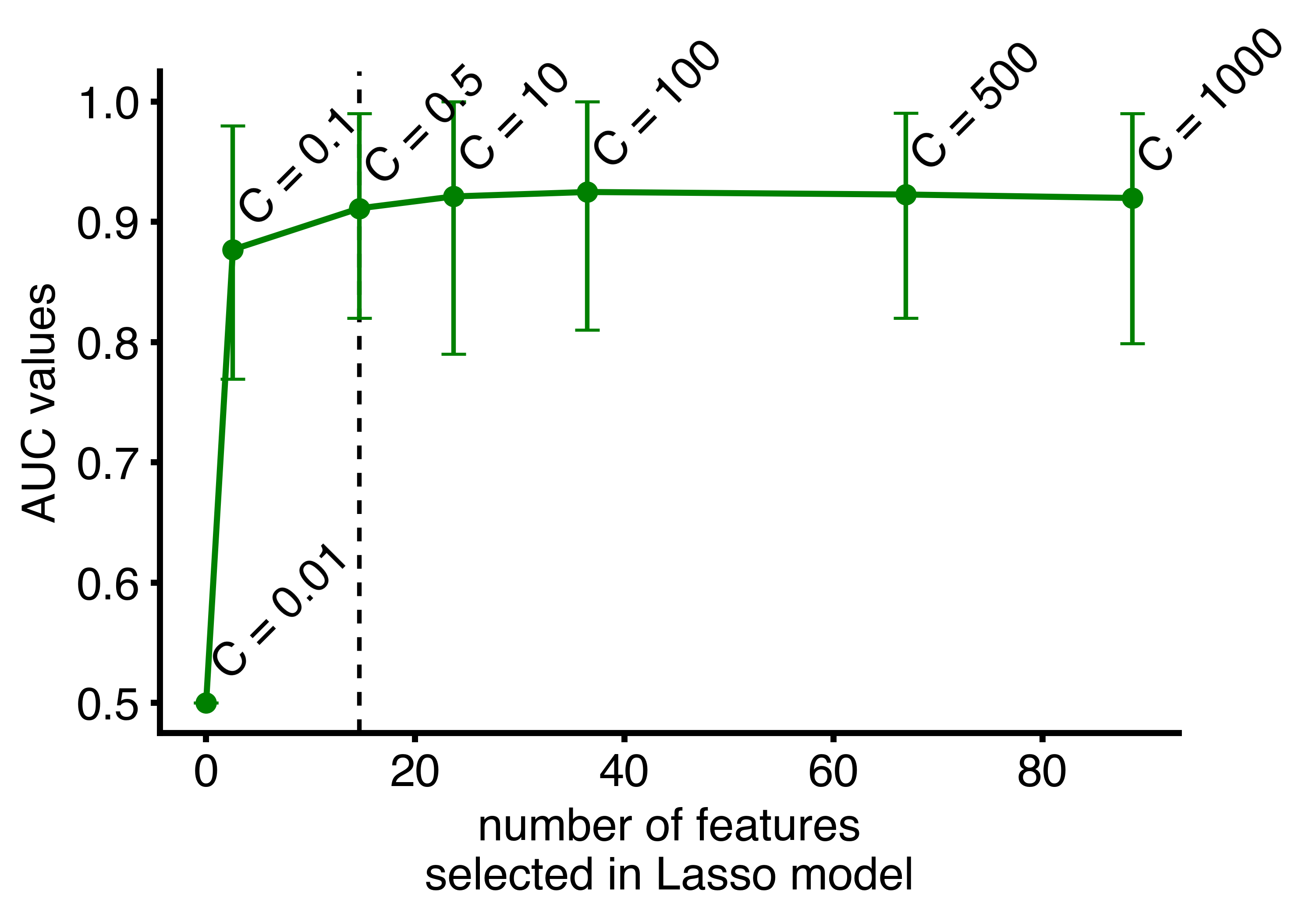


**Figure S2.** The area under the receiver-operator curve (AUC) values estimated for Lasso logistic regression models (python sklearn.linear_model LogisticRegression with penalty='l1’) separating healthy controls from depression patients (*n* = 36 and *n* = 32, respectively), using 1000 random test-train splits and parameters C = 0.01, 0.1, 0.5, 10, 100, 500 1000, plotted against the average number of features selected by the Lasso logistic regression model.


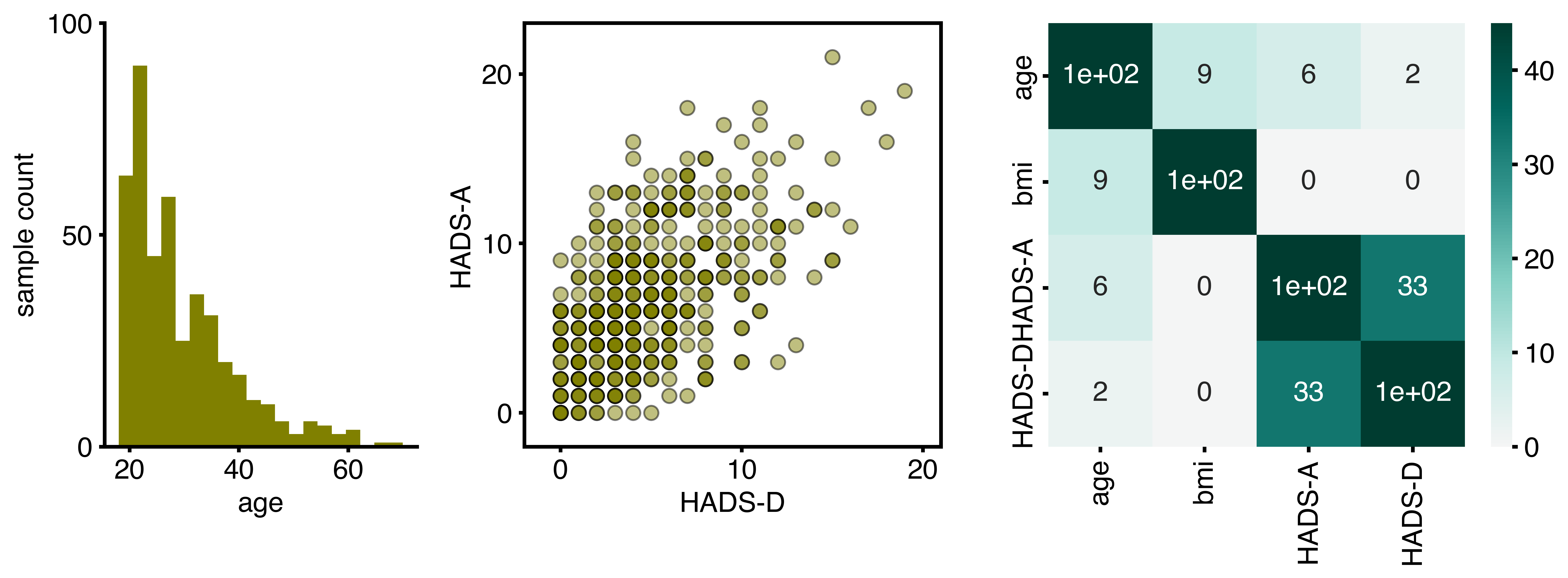


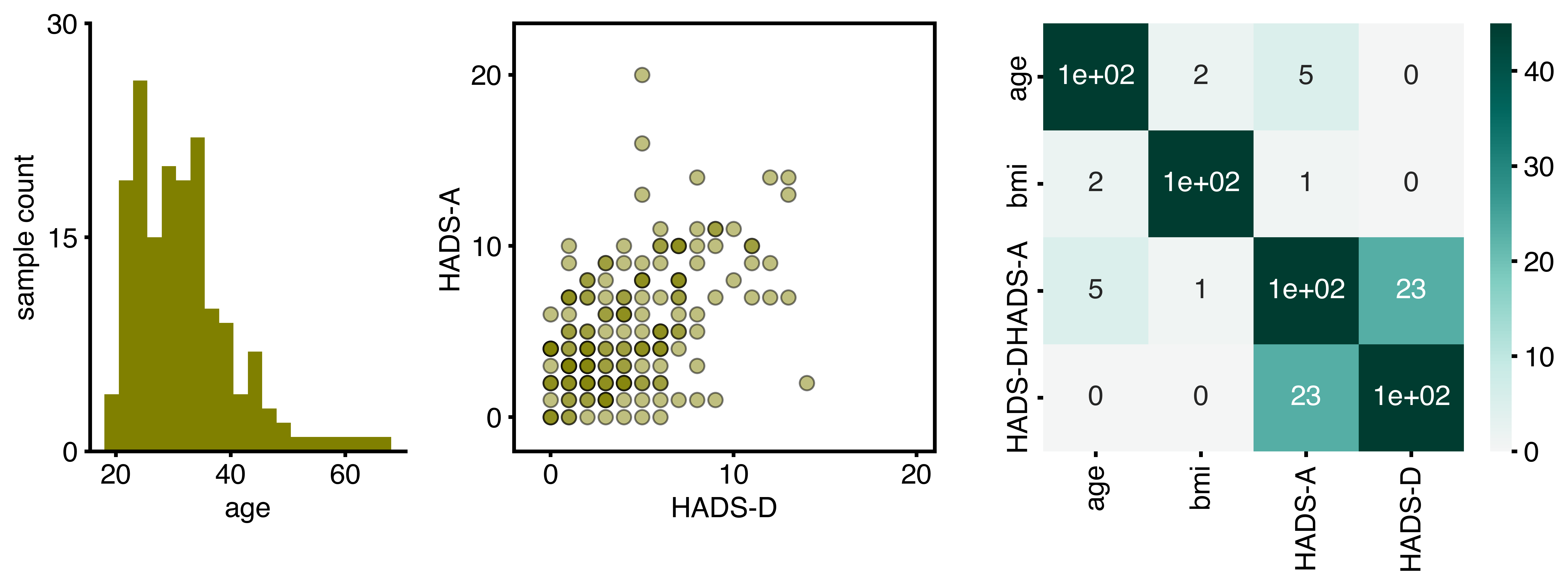


**Figure S3.** Demographic and mental health characteristics of the volunteer cohort, top: females (*n* = 437), bottom: males (*n* = 167). (Left) The sex distribution among the volunteers in the cohort. (Middle) The distribution of self-reported anxiety (HADS-A) and depression (HADS-D) symptom scores among the individuals in the cohort. (Right) The co-dependency among demographic factors and self-reported clinical indicators. The numbers depicted represent the percentage of variation (R2) explained for one specific variable by another variable, as determined by linear regression model analysis.


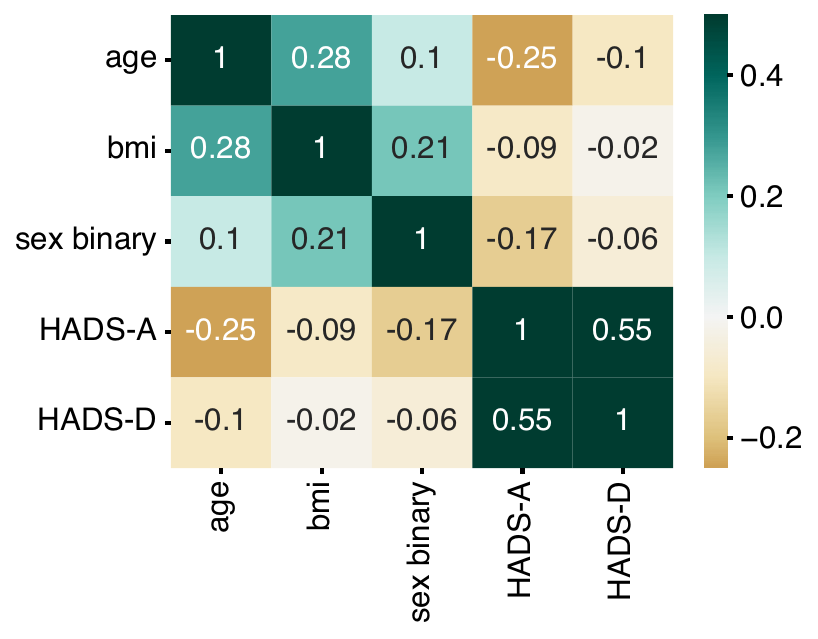


**Figure S4.** The correlation among demographic factors and self-reported HADS scores (*n* = 604). The depicted numbers represent the Pearson correlation coefficients between variables.


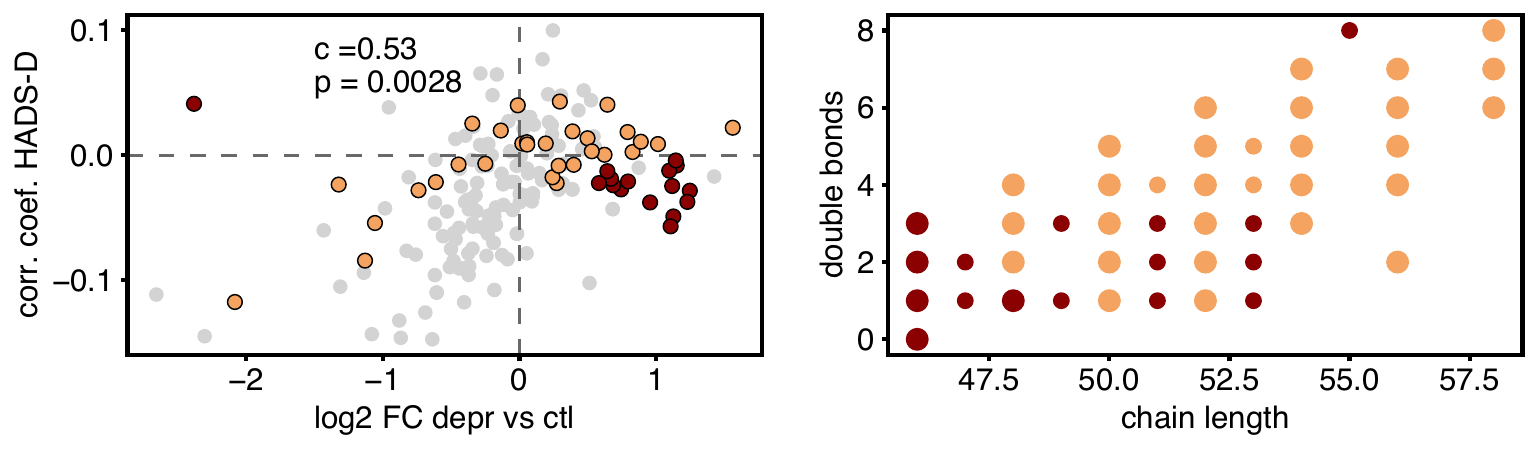


**Figure S5.** Shorter-chained and more saturated triglycerides show stronger discrepancy between high HADS-D volunteers and clinical depression patients. Left: relationship between the association of HADS-D and lipid abundances (Pearson correlation coefficients) and alterations in lipid abundances in clinical depression (mean base-2 log transformed fold-changes between clinical depression and the volunteer cohort). Triglycerides with corresponding absolute mean log2 FC > 0.5 and opposing directions of effects are colored in dark red, the rest are colored in orange. The Spearman correlation coefficient and p-value is indicated for the latter group of triglycerides (*n* = 30 lipids). Right: the same two group of triglycerides, for which total chain length and double bonds are depicted. Larger marker sizes correspond to even-chain lipids.


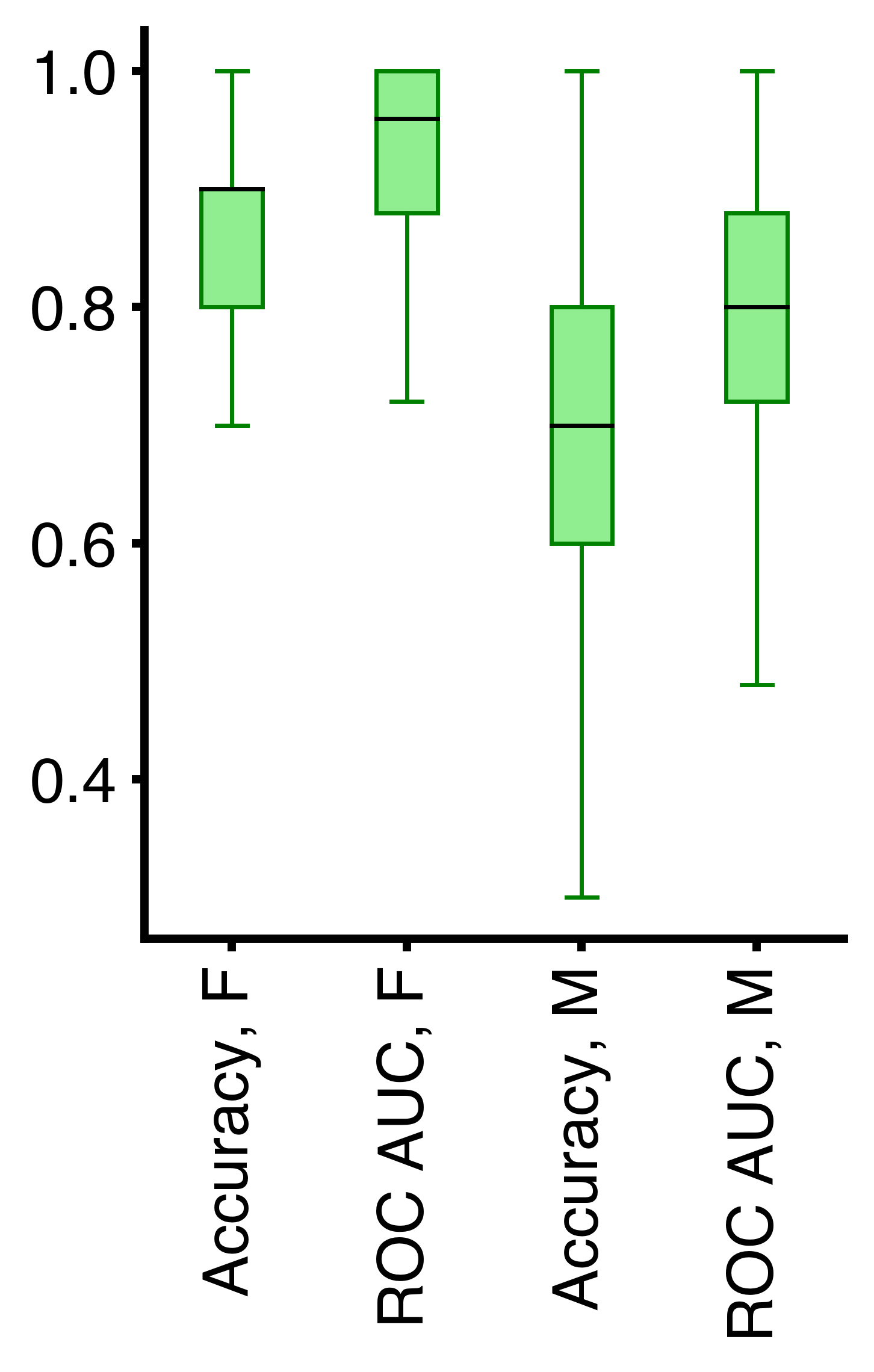


**Figure S6.** Predictive modeling for the detection of individuals with high HADS-D scores. Boxplots illustrate the randomized cross-validation accuracy and ROC AUC values of the model separating clinical depression from healthy controls for females (F, *n* = 15 and 23) and males (M, *n* = 17 and 13) separately. The train-test split for randomized cross-validation was performed as follows: 5 female/male patients with clinical depression and 5 female/male controls were used in testing, and 16 sex-balanced individuals with clinical depression and 16 sex-balanced controls were used in training.


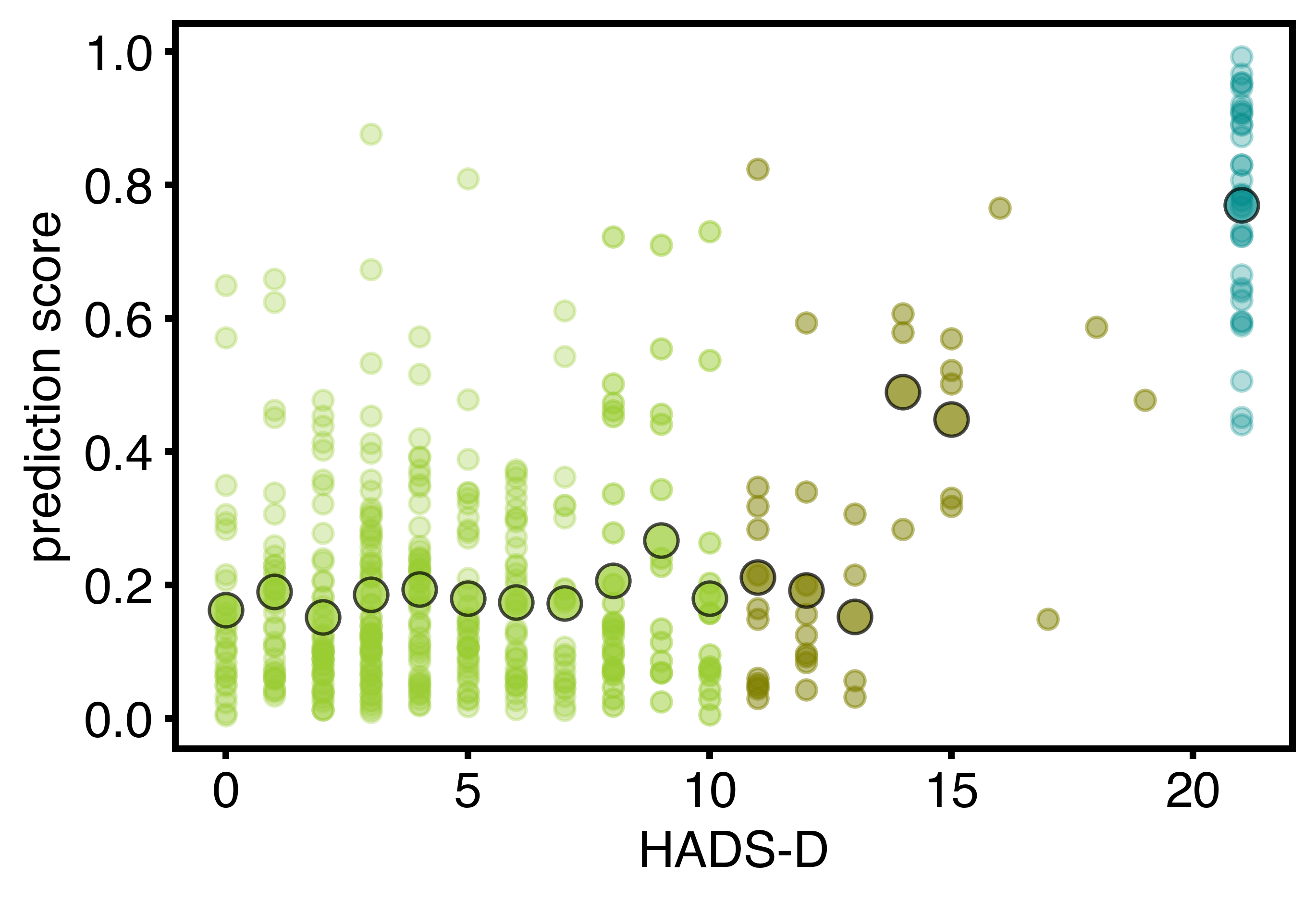


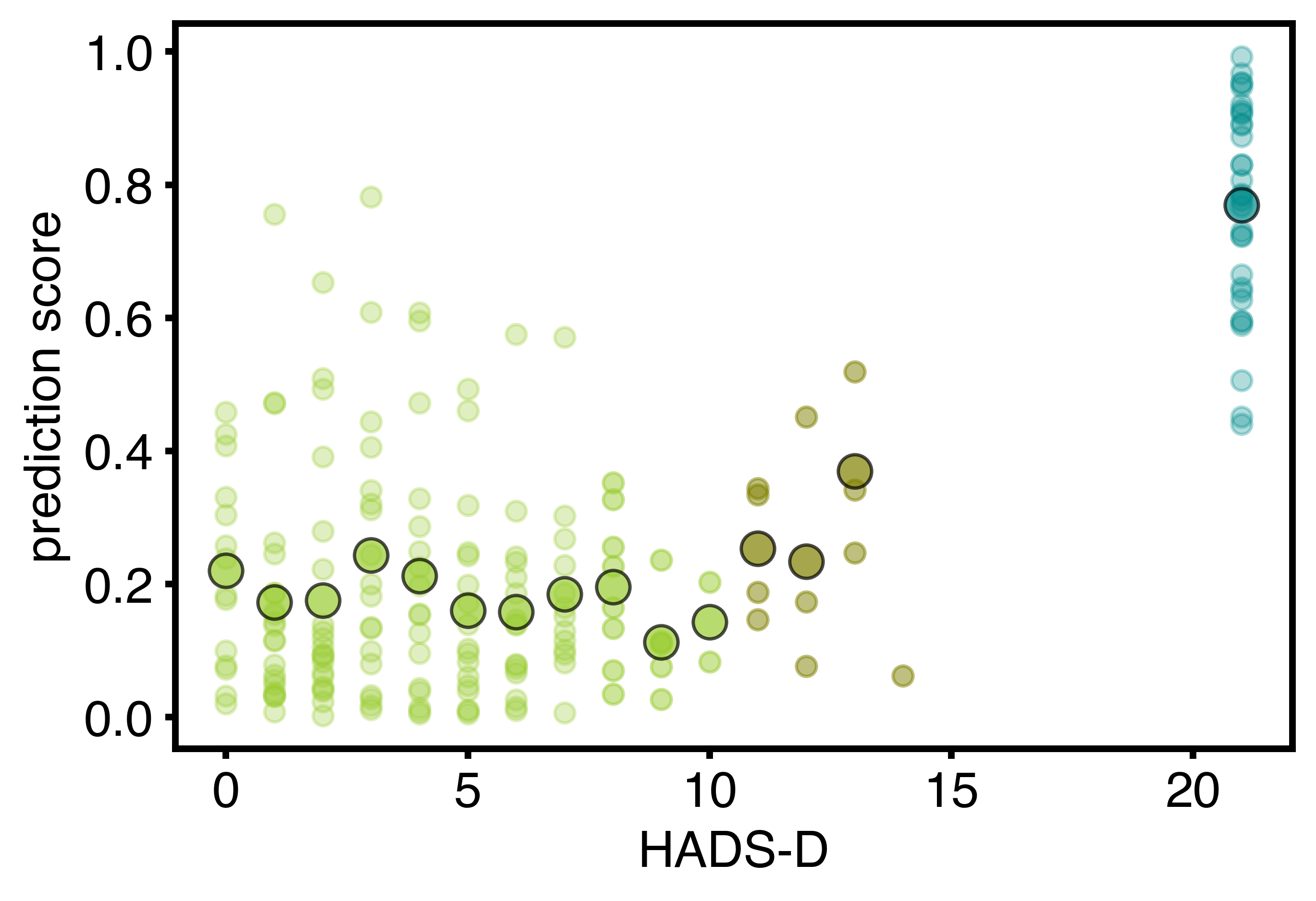


**Figure S7.** The correlation between volunteers' HADS-D values and their predicted scores derived from the model trained on clinical depression patients versus controls, top: only females volunteers (*n* = 425, excluding 12 volunteers used in training), bottom: only male volunteers (*n* = 164, excluding 3 volunteers used in training). Individual predictions are represented by colored points, with mean predicted scores for each HADS-D value indicated by larger circles. Light green represents volunteers with no or mild symptoms (HADS-D = 0 - 10), dark green represents those with moderate to severe symptoms (HADS-D ≥ 11), and teal blue symbols on the right represent clinical depression patients (both sexes). Top (females): Spearman correlation *c* = 0.2, *p* < 0.0001 for all values (*n* = 425); Pearson correlation *c* = 0.61, *p* = 0.0013 for averaged prediction scores across discrete HADS-D values (*n* = 16). Bottom (males): Spearman correlation *c* = 0.015, *p* = 0.85 for all values (*n* = 164); Pearson correlation *c* = 0.33, *p* = 0.25 for averaged prediction scores across discrete HADS-D values (*n* = 14).


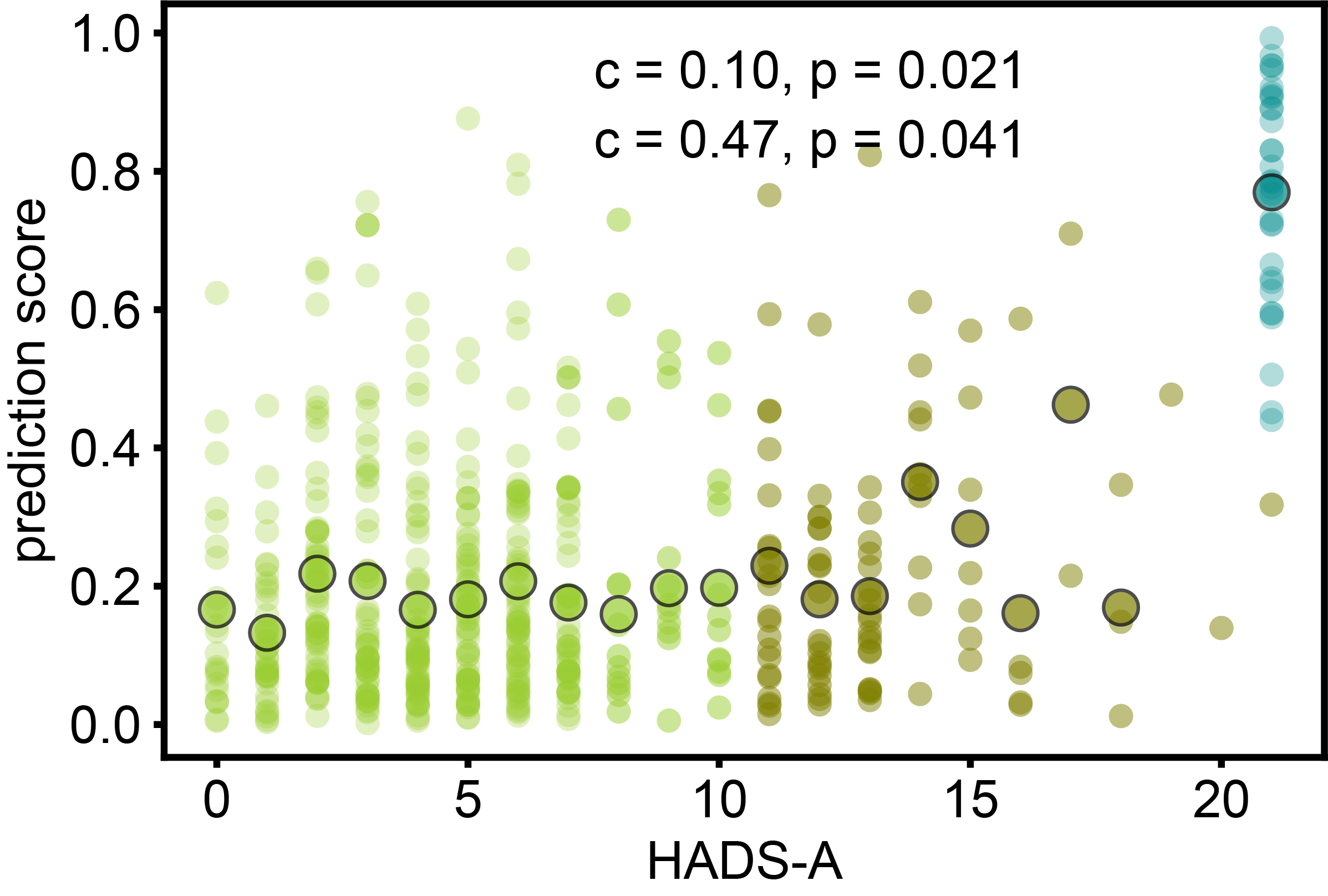


**Figure S8.** The relationship between volunteers' HADS-A values and their predicted scores derived from the model trained on clinical depression patients versus controls (*c* and *p* on the top: Spearman correlation coefficients and p-values, *n* = 589, excluding 15 volunteer individuals used in model training). Individual predictions are represented by colored points, with mean predicted scores for each HADS-A value represented by larger circles (*c* and *p* below: Pearson correlation coefficients and p-values for averaged prediction scores across discrete HADS-A values, *n* = 19). Light green illustrates volunteers with no or mild symptoms (HADS-A= 0 - 10), dark green illustrates those with moderate to severe symptoms (HADS-A ≥ 11), and teal blue symbols on the right illustrate clinical depression patients.


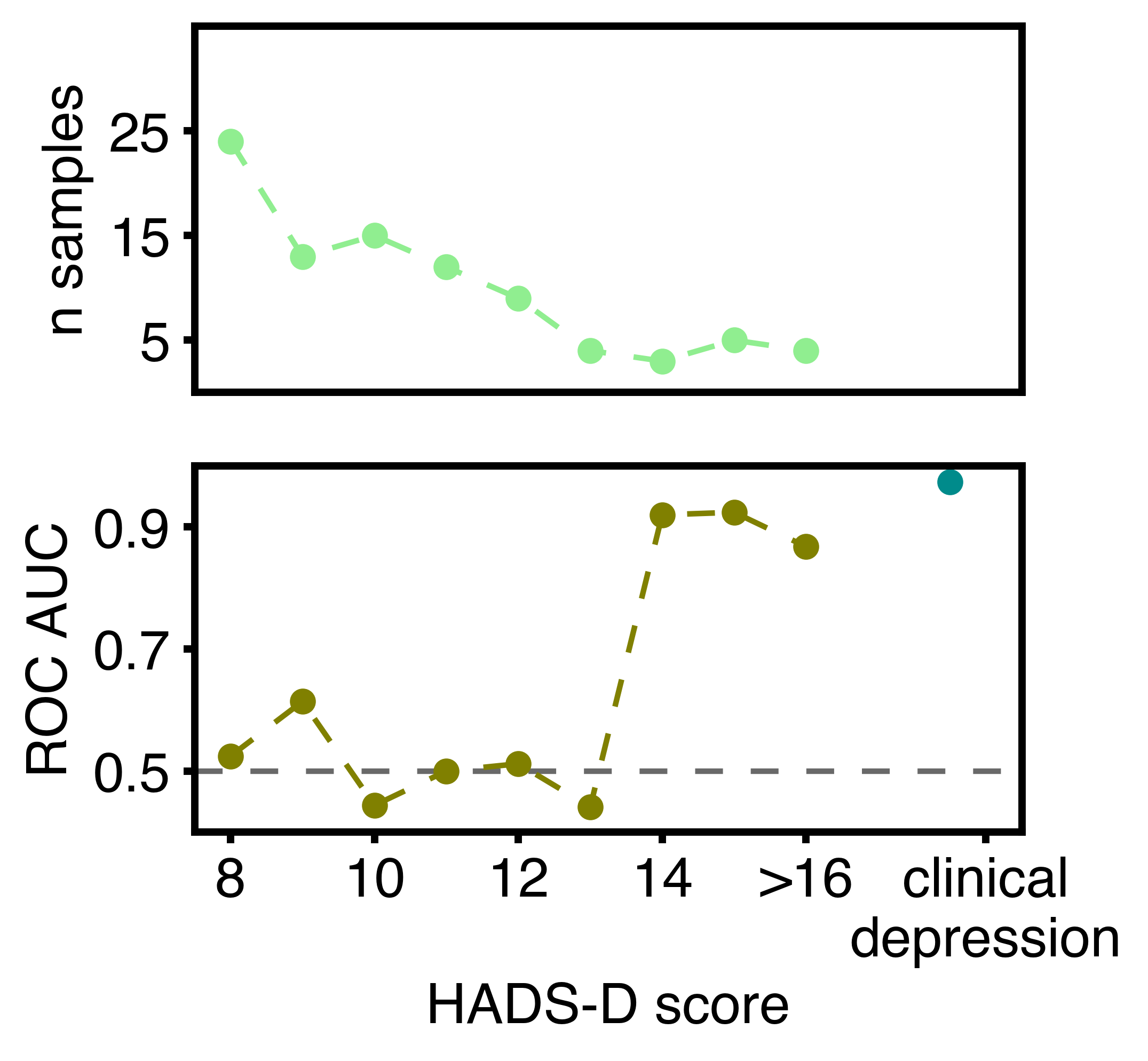

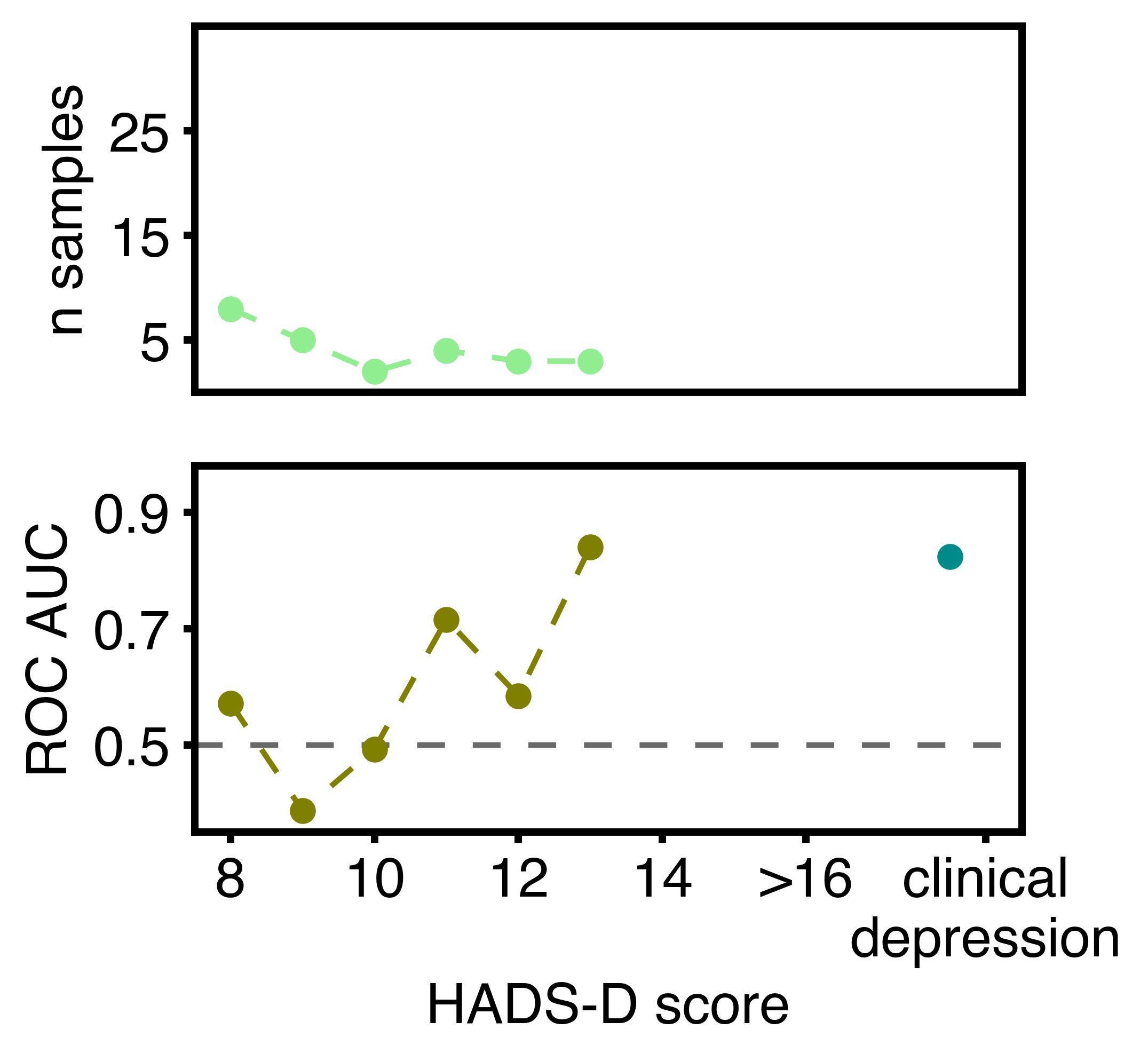


**Figure S9.** Predictive modeling for the detection of individuals with high HADS-D scores, for females (left) and males separately (right).Top: the number of individuals with specific HADS-D scores. Bottom: ROC AUC values of the model performance in distinguishing individuals with the specific HADS-D scores (dark olive), as well as clinical depression (teal blue), from those without depressive symptoms (HADS-D ≤ 7).
